# Heavy metal pollution and risk assessment by the battery of toxicity tests

**DOI:** 10.1038/s41598-020-73468-4

**Published:** 2020-10-06

**Authors:** Mohd. Shahnawaz Khan, Mehjbeen Javed, Md. Tabish Rehman, Maryam Urooj, Md. Irshad Ahmad

**Affiliations:** 1 Protein Research Chair, Department of Biochemistry, King Saud University, Riyadh, Saudi Arabia; 2 Department of Zoology, Aligarh Muslim University, Aligarh, U.P. India; 3 Department of Science, T.R. Kanya Mahavidyalaya, Aligarh, U.P. India; 4 Department of Pharmacognosy, College of Pharmacy, King Saud University, Riyadh, 11451 Saudi Arabia; 5 Unaffiliated, Yanbu, Saudi Arabia; 6 Department of Biochemistry, Faculty of Life Sciences, Aligarh Muslim University, Aligarh, U.P. India; 7 Department of Biophysics, All India Institute of Medical Sciences, New Delhi, India

**Keywords:** Environmental impact, Biochemistry, Zoology

## Abstract

The current study was carried out on dominant fish *Oreochromis niloticus* and water collected from the polluted Yamuna River, Agra, India. The heavy metals in water, recorded as follows: Fe > Mn > Zn > Cu > Ni > Cr > Cd and all were found to be above the prescribed limits. According to metal pollution index, exposed muscle (49.86), kidney (47.68) and liver (45.26) have been recorded to have higher bioaccumulation. The blood biochemical analysis of exposed *O. niloticus* indicated significant increase in activities of aspartate aminotransferase (+ 343.5%), alkaline phosphatase (+ 673.6%), alanine aminotransferase (+ 309.1%), and creatinine (+ 494.3%) over the reference. However, a significant decrease in albumin (A): globulins (G) ratio (− 87.86%) was observed. Similarly, the exposed fish also showed significant increase in total leucocyte count (+ 121%), differential leucocyte count, respiratory burst (+ 1175%), and nitric oxide synthase (+ 420%). The histological examination of liver and kidney showed tissue injury. Moreover, micronuclei (0.95%), kidney shaped nuclei (1.2%), and lobed nuclei (0.6%) along with DNA damage in the form of mean tail length in the liver (20.7 µm) and kidney (16.5 µm) was observed in the exposed *O. niloticus*. Potential health risk assessments based on estimated daily intake, target hazard quotient, hazard index, and target cancer risk indicated health risks associated with the consumption of these contaminated fishes. In conclusion, the present study showed that exposure to heavy metals contaminated water can alter immunological response; induce histopathological alterations and DNA damage in the studied fish. The consumption of this contaminated water or fish could have serious impact on human health.

## Introduction

Pollutants (heavy metals, pesticides, PAHs etc.) are released into the environment by anthropogenic activities. Humans use water bodies as sink for all their waste disposals, due to which quality of aquatic ecosystem is degrading day by day. This results in to less diversity of aquatic species and low quality of food from these resources^1^. Heavy metals are discharged into the environment after used in industries, factories, domestic wastes and leakage from the dump sites^2,3^. These metals reach to the environment such as air, water, soil and food in different forms. In spite of their low concentrations in the different surface waters, they show high bioaccumulation in fishes and other fauna and flora of water bodies^4^. Hence, fish is good experimental model to assess the adverse effects of heavy metals. Turan et al.^5^ took *Clarias gariepinus* as a bioindicator of Orontes River, Turkey to highlight the degraded quality of water and adverse effects of heavy metals (Cr, Cd, Cu, Fe and Mn) in terms of bioaccumulation, oxidative stress and genotoxicity. Ahmed et al.^6^ studied the bioaccumulation of heavy metals (As, Pb, Cd, Cr and Cu) in different species of fish *Apocryptes bato*, *Pampus chinensis*, *Liza parsia*, *Mugil cephalus*, *Hyporhamphus limbatus*, *and Tenualosa toil*, since they all are consumed by locals of Bangladeshi population and reported six-times higher non-carcinogenic risk in children. Maurya et al.^7^ studied bioaccumulation and human health risk due to Zn, Pb, Cu, Cd, and Cr pollution in *Cirrhinus mrigala*, *Cirrhinus reba*, *Catla catla*, *Labeo rohita*, *Crossocheilus latius*, *Clupisoma garua*, and *Mystus tengara* collected from Ganga river basin.

The present investigation chose most abundant fish *Oreochromis niloticus* (nile tilapia) and studied several important enzymes of liver and kidney such as aspartate aminotransferase (AST), alkaline phosphatase (ALP), alanine aminotransferase (ALT) and creatinine (CK). These enzymes get released into the circulation as a result of some pathologies^8^. Their innate immunity which is considered as the main defense system of fish was also studied^9^. Few authors also considered, fishes as the important model for non-specific/innate immunity and ecotoxicology^9,10^. Immuno-competence is indispensable for the maintenance of the complete health of living being and is immensely attuned to toxins, like heavy metals^10,11^. Researchers also suggested that damage of innate immunity may be more remarkable in fish compared to mammals^9^. Respiratory burst assay is as an indicator of the innate immune system which releases cytokines and inflammatory response in fish. During respiratory burst process, the phagocytes and leukocytes of pathogens increase their oxygen consumption with the help of NADPH oxidase enzymes and generate various reactive oxygen specie such as singlet oxygen (^1^O_2_), superoxide anion radical (O_2_), hydroxyl radical (OH^−^), and hydrogen peroxide (H_2_O_2_) to be used as a defense mechanism against toxic agents^10,12^. In addition, the nitric oxide synthase (NOS), the total leukocyte count (TLC), the differential leukocyte count (DLC), and the globulin concentrations are also served as good immunological index^12^. The heavy metals are known to cause histopathology and genotoxicity. Histopathology of the target organs such as liver and kidney could give clear picture of toxicity of the heavy metals. Among genotoxicity assays micronuclei and single cell gel electrophoresis (SCGE) are the rapid and more reliable tools for the ecotoxicological evaluations^8,13,14^. Besides, some populations, chiefly those who take large amounts of fishes, can accumulate concentrations which are deleterious to their health as well as their children^15^. The present study aims to discuss the toxicities of heavy metals on the marker enzymes, immunotoxicity, histopathology and genotoxicity in freshwater tilapia fish, *Oreochromis niloticus*. The human health risk assessment such as estimated daily intake (EDI), non-carcinogenic (THQ) and carcinogenic risk (TR) and hazard index (HI) are also calculated.

## Results and discussion

### Bioaccumulations and MPI value in muscle, liver, and kidney

The water quality parameters of Yamuna river (sampling site of studied fish *O. niloticus*) such as temperature (27.5 °C), pH (7.4) and D.O (6.3 mg/L) were found to be within ideal standards of Bureau of Indian Standards (BIS)^16^ and WHO (adapted from UNEPGEMS)^17^. On the other hand, the heavy metal proportion was in the order of Fe (48 mg/L) > Mn (19 mg/L) > Zn (13 mg/L) > Ni (11.9 mg/L) > Cu (7 mg/L) > Cr (3.5 mg/L) and all were found to be beyond the prescribed standards of BIS and WHO^16,17^. At reference site, the temperature (27 °C), pH (7.5), D.O (6.5 mg/L) were also found to be ideal. Furthermore, the heavy metals concentration were within the prescribed limits and were present as follows: Fe (5.2 mg/L) > Zn (3.9 mg/L) > Cu (3.6 mg/L) > Mn (0.02 mg/L). While Ni and Cr were found to be below the detection limits of atomic absorption spectrophotometer. The bioaccumulation was studied in muscle, liver, and kidney of *O. niloticus* (Table 1). In exposed fish, Cu and Mn showed the highest and lowest accumulation in muscle and liver as compared to respective reference fish respectively. The Ni exhibited maximum concentrations in kidney followed by liver and muscle of exposed *O. niloticus*. The MPI assessment in exposed fish showed muscle as target organ followed by kidney and liver, whereas, in reference fish’s the trend was muscle > liver > kidney (Table 2). It shows that *O. niloticus* muscle have more capability of heavy metal accumulation and have no or less active mechanism for their discharge. Similar bioaccumulation of As, Cd, Pb, and Hg have been reported in *P. fulvidraco*, *C. auratus*, and *H. nobilis* collected from Nansi Lake of China^18^. In a recent study, accumulation of As, Pb, Cd, Cr, and Cu in six edible fish species was studied where Cd, Pb and Hg were suggested as highly deleterious heavy metals accompanied by Cu, Cr, Ni, Mn, and Zn and their high solubility produce threat to inhabiting fish^6^. Prolonged exposure to even very low amounts of heavy metals could lead to leakage of pathology marker enzymes, immunotoxicity, and genotoxicity in different tissues of fish^19^. Hence, regular monitoring is required to confirm the adverse effects associated with these heavy metals.

**Table 1:** Heavy metal concentrations in fish tissues (mg/kg. dw).

	Cr	Mn	Fe	Ni	Cu	Zn
Exposed O. *niloticus*						
Muscle	_a_36.75 ± 0.72^e^	_b_9.8 ± 0.11^f^	_a_54.55 ± 1.2^b^	_c_34 ± 0.3^d^	_a_296.05 ± 1.8^a^	_a_77.7 ± 0.96^c^
Liver	_a_30.75 ± 0.53^e^	_a_20.2 ± 0.31^f^	_b_38.9 ± 1.8^d^	_b_51 ± 0.47^c^	_b_93.65 ± 0.01^a^	_ab_74.55 ± 0.32^b^
Kidney	_b_25.55 ± 0.16^d^	_a_19.4 ± 0.01^e^	_b_41.9 ± 0.18^c^	_a_91.3 ± 0.3^a^	_b_89.7 ± 0.2^a^	_b_69.15 ± 0.2^b^
Reference *O. niloticus*						
Muscle	–	_c_3.6 ± 0.1^d^	_cd_10.7 ± 1.3^c^	–	_c_17 ± 0.001^a^	_cd_13.9 ± 0.87^b^
Liver	–	_cd_2.8 ± 0.1^c^	_c_15.93 ± 1.9^a^	–	_d_6.6 ± 0.21^b^	_c_16.9 ± 0.5^a^
Kidney	–	_de_2 ± 0.01^d^	_d_8.4 ± 0.01^b^	–	_d_5.9 ± 0.01^c^	_d_12 ± 0.11^a^

All values are given as mean ± SEM (n = 15); Two way ANOVA and DMRT was used for statistical analysis. Means with similar letters (superscripts and subscripts) along the column and row are statistically similar at *p* < 0.05; Blank cells indicate below detection limits.

**Table 2:** MPI values in both group of fishes.

Fish groups	Tissues	MPI
Exposed *O. niloticus*	Muscle	49.86
	Liver	45.26
	Kidney	47.68
Reference *O. niloticus*	Muscle	9.76
	Liver	8.39
	Kidney	5.87

### Pathology marker enzyme activities

Enzymes are widely employed as biomarkers to assess physiological changes in organisms as a result of environmental toxicants. Heavy metals are known to have impact on enzyme activities, which would have involvement in specific substrate availability for metabolism^20^. In this study, serum ALP (+ 673.6%), ALT (+ 309.1%), AST (+ 343.5%) and CK (+ 494.3%) activities were found to be comparatively higher in exposed *O. niloticus* as compared to reference fish (Table 3). The rise in these enzymatic activities could be due to lesions in liver and kidney in response to bioaccumulation. Recently, Barisic et al.^21^ observed exposure period related activities of AST, ALT and ALP in serum of salmonid. They observed that higher activities of these enzymes leading to more liver damage. Enzymes like ALT, AST, and ALP are the most frequently used serum markers to elucidate injury in liver hence they are called serum aminotransferases^22^. Few researchers reported elevated levels of serum AST, ALT, and ALP in *Cyprinus carpio* and *Channa punctatus* in response to heavy metals exposure^23,24^. Moreover, the higher level of CK indicates kidney damage due to heavy metals^25^. A marked alteration in the osmoregulatory behavior because of kidney damage was eported by Schjolden et al.^26^ as a result of Cu exposure. However, Barisic et al.^21^ and Banaee et al.^27^ suggested this enzyme as marker to both liver and kidney function. CK employ adenosine-triphosphate to catalyze the transformation of creatine into adenosine diphosphate and phosphocreatine. The higher the CK activity higher the tissue damage causing acute kidney injury, rhabdomyolysis, muscular dystrophy, myocardial infarction and autoimmune myositis^28,29^.

**Table 3:** Pathological marker enzymes and immune parameters in serum/blood.

Variables	Reference *O. niloticus*	Exposed *O. niloticus*	% change over reference
AST (U/L)	4.87 ± 0.19	21.6 ± 0.21*	+ 343.53
ALT (U/L)	8.31 ± 0.33	34 ± 1.5*	+ 309.14
ALP (U/L)	7.2 ± .67	55.7 ± 4.5*	+ 673.61
CK (U/L)	10.6 ± 1.0	63 ± 3.9*	+ 494.33
Alb (mg/dl)	1.8 ± 0.11	0.92 ± 0.09*	− 48.88
Glo (mg/dl)	0.87 ± 0.08	3.7 ± 0.11*	+ 157.22
A/G ratio	2.06 ± 0.18	0.25 ± 0.03*	− 87.86
TLC (10^3^/mm^3^)	29.4 ± 3.2	65 ± 2.8*	+ 121.08
Neutrophils%	27 ± 2.6	89 ± 4.5*	+ 229.62
Lymphocytes%	33 ± 5.4	70 ± 6.7*	+ 112.12
Eosinophils%	1.7 ± 0.2	8.4 ± 0.15*	+ 394.11
Basophils%	0 ± 0.0	10 ± .9*	–
Monocytes%	16.4 ± 3.3	28 ± 2.9*	+ 70.73
Respiratory burst	1.2 ± 0.24	15.3 ± 0.16*	+ 1175
NOS (mol/mL)	5 ± 0.9	26 ± 2.2*	+ 420

All values are given as mean ± SEM (n = 15); t-test was used for statistical analysis; Asterisk indicates significant difference at *p* < 0.05; + or – indicates increase or decrease over reference.

### Serum albumin (A) and globulins (G)

Albumin and globulin makes most of the proteins and any modification in their amounts results in disturbance of A:G ratio^8^. Influence of heavy metals has also been noticed on serum albumin and globulin levels. In the present study, lower albumin (− 48.88) and excessive globulin (+ 157.22) amount was observed in the exposed fish compared to reference *O. niloticus*, consequently leading to lower A:G ratio in exposed *O. niloticus* (0.25) than reference *O. niloticus* (2.06) (Table 3). Corroborating results has been reported in *M. cavasius* fish collected from water polluted from electroplating industry effluents^30^. Recently, Barisic et al.^21^ also reported a significant decrease in serum total protein and albumin parameters in *Onchorhynchus mykiss*. The normal value range of A:G ratio should be in between 0.8 and 2.0, which helps in indexing to monitor differences in the framework of serum or plasma^24^. The lower A:G ratio in exposed *O. niloticus* is the indicative of higher liver damage.

### Innate immunity

Effects of heavy metals on pathological marker enzymes and immune parameters of serum/blood are shown in Table 3. Total leukocyte count (TLC) is an important parameter to assess the stress^31^. Leucocytes represent a major component of body’s defense against foreign bodies, infections, and toxins. A significant increase in TLC (+ 121.08) level was observed as compared to reference *O. niloticus* (Table 3). This increase in TLC by heavy metal overload could be associated to a stimulation of immune response due to tissue damage^32^. Fish blood mainly contains neutrophils, lymphocytes, and monocytes whereas, eosinophils and basophils are rarely observed or may even be absent in healthy conditions^33^. In exposed fish, the percentage of neutrophils (+ 229.62%), lymphocytes (+ 112.12%), eosinophils (+ 394.11%) and monocytes (+ 70.73) were found to be significantly higher when compared to the reference fish (Table 3). This result reflects prominent immune response in exposed *O. niloticus*. Higher lymphocytes in treated fish as compared to reference may be attributed to considerable action of B-cells, T-cells, and natural killer cells, which react to identify antigens, produce antibodies and wipeout the target cells causing damage. A significant correlation between heavy metals and proliferation of lymphocytes was reported by Lawrence^34^. On the contrary, higher neutrophil percentage may be due to the action of myeloperoxidase enzyme. Myeloperoxidase is abundantly present in neutrophil cells and eliminate invaders from the body of the fish^8^. Further, immune reciprocation was checked by respiratory burst and NOS enzyme activity. Elevation in respiratory burst (+ 1175%) in exposed *O. niloticus* than reference fish indicates heightened phagocytic activity to counter the toxic agents (Table 3). Other researchers have also reported higher respiratory burst against variety of foreign agents^35,36^. A significant higher level of NOS (+ 420%) was also observed in exposed *O. niloticus* than reference group (Table 3). NOS catalyzes the generation of cellular signaling molecule nitric oxide that participate crucially in defense mechanism of fish^12,37^. Thus, the elevated levels of respiratory burst activity and NOS indicated the infection or injury of *O. niloticus.*

### Histopathology

**Figure 1:**
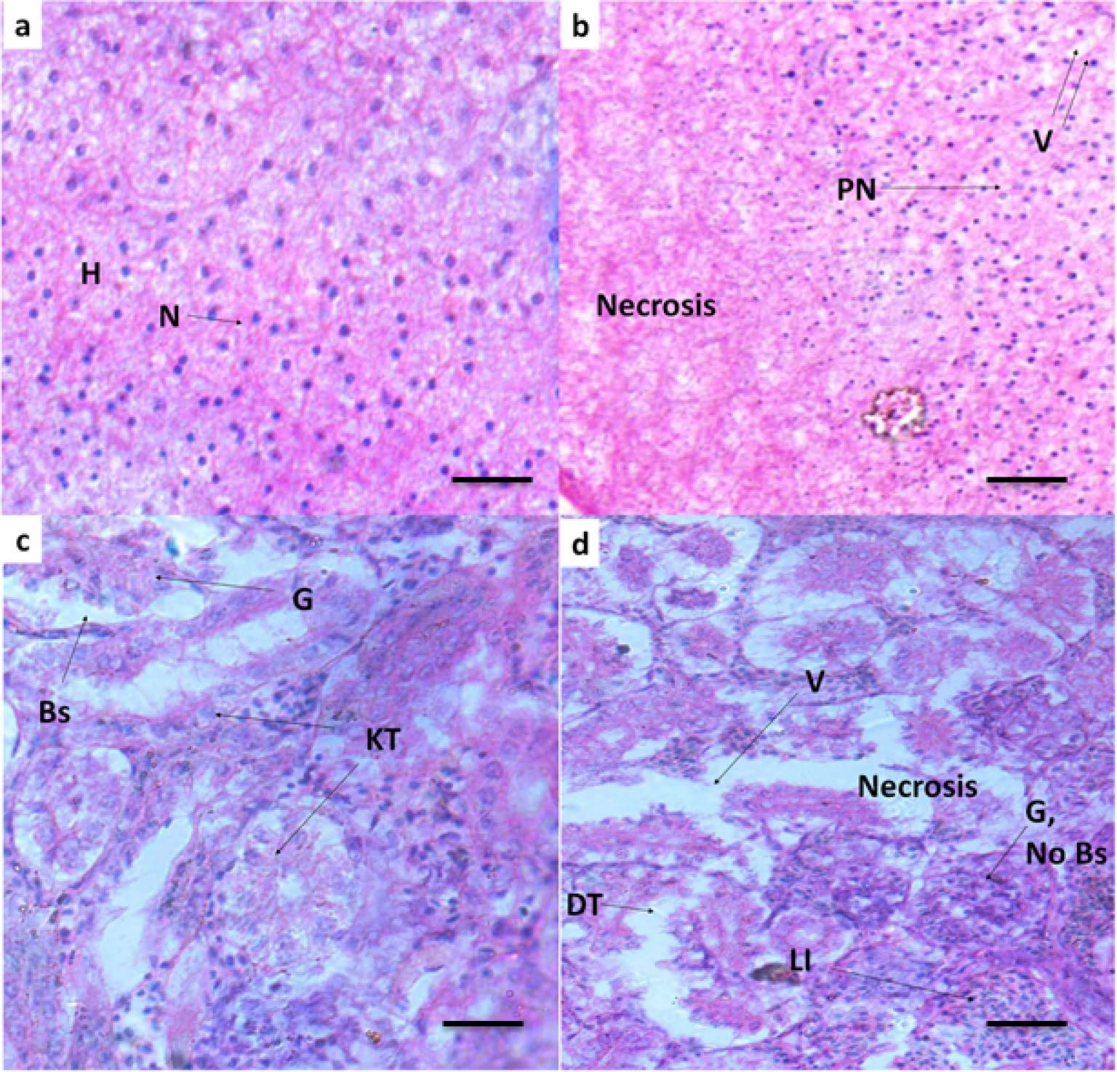
Histopathology of liver and kidney of *O. niloticus* inhabiting in reference and polluted water. (**a**) Reference liver; (**b**) Liver of exposed fish; H (hepatocyte), N (Nucleus), Necrosis, V (vacuolization). (**c**) Reference kidney; (**d**) Kidney of exposed fish; G (glomeruli), Bs (bowman's space), KT (kidney tubule), DT (damaged tubule), Necrosis, LI (Leucocyte infiltration). Magnification = 40X; Scale bar = 20 µm.

The histopathological examinations of liver and kidney of reference and exposed *O. niloticus* are shown in Fig. 1. In liver tissue, reference fish showed normal hepatocytes and nucleus (Fig. 1a) whereas in exposed fish congestion of blood vessel, vacuolation, pyknotic nuclei, and necrosis were observed (Fig. 1b). These observations suggest that excess of heavy metals caused histological alterations mainly via generations of reactive oxygen species^38^. Hepato-cellular damage, caused by heavy metal overloads has also been reported earlier in other fish species^24,39^. Kidney sections of the reference group shown in Fig. 1c, showed a normal structure such as glomeruli, bowman’s space with uniform kidney tubules. However, the major hallmarks that were observed in exposed fish tissue were decreased or no bowman’s space, large vacuolation, necrosis, damaged tubule and glomeruli, and leucocyte infiltrations (Fig. 1d). Several other studies also reported the abnormalities in kidney structure in other fishes due to the exposure of metal contaminated water in the form of reduction of renal hematopoietic system, tissue damage, necrosis, glomerular injury, proliferation of connective tissue, glomerular and epithelial tubuli contraction^39,40^.

### Micronucleus test

**Figure 2:**
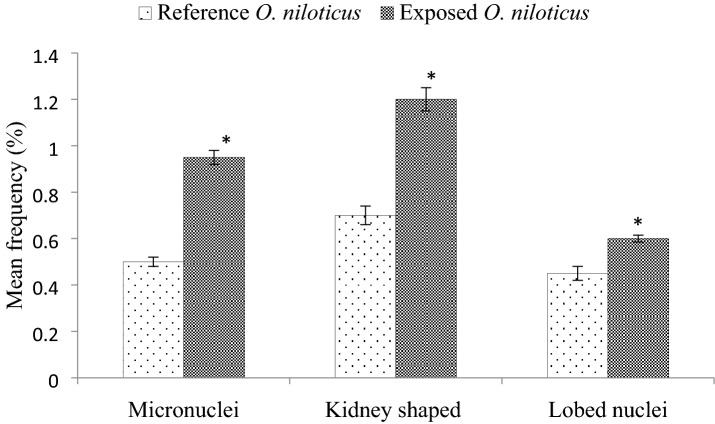
Mean frequencies (%) of micronuclei, kidney shaped and lobed nuclei in erythrocytes of reference and exposed *O. niloticus.* t-test was used for statistical analysis, significance level was established at *p* < 0.05.

Micronuclei are produced by the centric chromosomal fragments during anaphase stage of cell division. Heavy metals inducing such deformities in fishes as reported earlier too^41,42^. The observed nuclear abnormalities in erythrocyte of *O. niloticus* were kidney shaped nuclei, lobed nuclei and micronuclei (Fig. 2). Most significantly detected abnormalities in the exposed *O. niloticus* were micronuclei (0.95%) and kidney-shaped nuclei (1.2%) while the least frequent deformity was lobed nuclei (0.6%) as compared to reference fish (0.5%, 0.7%, and 0.45%) respectively (Fig. 2). Several investigators also reported that these nuclear abnormalities are caused by heavy metals which could lead to impaired DNA synthesis and DNA strands break^42,43^. During cell division, such toxicants can produce a mutation which may pass on to the future generations resulting in aneuploidy, diminish reproduction, reduces survival, and may also endanger the species^13,44^.

### DNA damage determination via single cell gel electrophoresis (SCGE)

**Figure 3:**
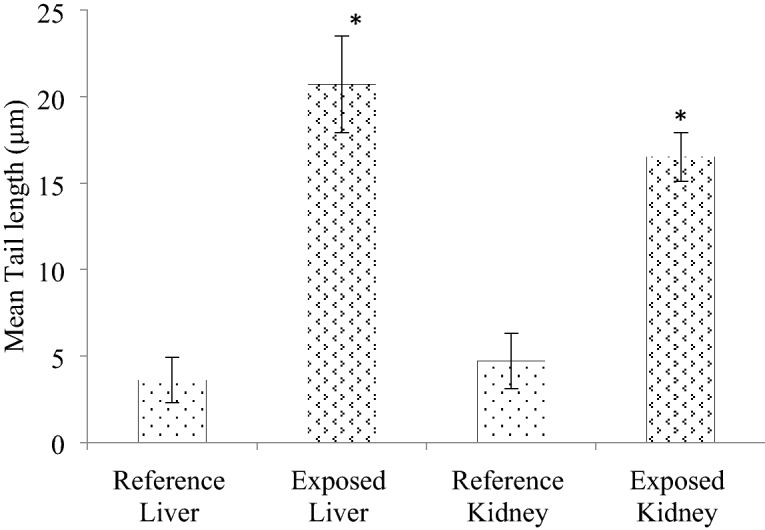
The effects of heavy metals on DNA damage in reference and exposed *O. niloticus* liver and kidney tissues. Data represented mean  ± SEM, (n = 6); t-test was used for statistical analysis and level of significance established at *p* < 0.05.

The SCGE assay is fast and sensitive technique for assessing the genotoxicity in terms of DNA damage caused by environmental pollutants. DNA plays crucial role in various life processes because it carries genetic information. Figure 3 shows the DNA damage in liver and kidney cells of reference and exposed *O. niloticus* respectively. In the liver of exposed fish, a significant mean tail length was observed 20.7 µm as compared to 3.6 µm in the reference. Similarly, in kidney, tail length of 16.5 µm was observed in exposed fish as compared to reference fish (4.7 µm). The percent change over reference in liver and kidney were + 475% and + 251% respectively. Furthermore, it shows liver as the target organ for genotoxicity. Besides, this also clearly indicates that these metals bind to the DNA and damage it. This can also be co-related with the generation of reactive oxygen species due to heavy metals which cause oxidative stress and in turn leading to lesions in DNA. Some studies also reported the higher DNA damage in different fish species such as *C. punctatus*, *O. niloticus, C. gariepinus, L. cephalus* due to the exposure of heavy metals^40,43,45^. It has been reported that heavy metals come in contact with DNA and form adducts by intercalating or covalently binding with the DNA molecule. These metals generate ROS which could also lead to single and double strand breaks^46,47^.

### Human health risk assessment

As stated by United State Environmental Protection Agency (USEPA), the human health risk assessment is the process to estimate the nature and probability of harmful health effects of chemicals in contaminated environmental media, now or in future to humans (https://www.epa.gov/risk_assessment/health-risk.htm). In the present investigation parameters used for human health risk assessment were EDI, THQ, HI, and TR via consumption of fish *O. niloticus* (Table 4). These guidelines for risk assessment were established by USEPA for the evaluation of prospective health risk caused by prolonged exposure of any chemical contaminant^48^. They get influenced not only by intake amount of toxicant but also depends upon the several factors such as average body weight, duration, exposure frequency, and oral reference dose (RfD). In this study, EDI calculations showed multi-fold higher values than the reference concentration. THQ values were highest for Cr (4190.78 × 10^–6^) followed by Cu > Ni > Zn > Fe > Mn for both adult male and female persons. Furthermore, higher HI was recorded for females (8485.68 × 10^–6^) than males (7443.54 × 10^–6^) (Table 4). The THQ should not go beyond 1, else it present non carcinogenic risks to the exposed population^49^. It must also be noted that THQ reflects the level of concern only not a degree of risk^50^. Fortunately, from this work none of the heavy metals showed THQ values > 1. In addition, THQ values of every examined heavy metal were found to be higher in females as compared to the males. This could be attributed to the dissimilarity in their average weight and lifespan. Minimum risks reveals, if the ratio of EDI (heavy metal)/RfD was equal to or less than the its RfD^51^. If it is > 1–5 times the RfD then low risk, if > 5–10 times the RfD then moderate risk, but if > 10 times the RfD then the higher risk. Here, for all the heavy metals this ratio was found to be more than 20 times and up to thousand times higher, pointing possible health hazard associated to the public. Since, fish fillet contain all the inspected heavy metals, so hazard index (HI) must be calculated which is numerically equal to addition of all the THQs. If HI value is greater than 1, means it is an alarm for public health safety^52,53^. Fortunately, HI values do not raise concern in the present investigation. As per USEPA, criteria Cr and Ni are among the potent carcinogens list^54^. In the current piece of work, both male and females were found to be more susceptible to carcinogenic risk posed by Cr than Ni (Table 4). However, the overall carcinogenic risk expected from both Cr and Ni in *O. niloticus* is low. Similarly, New York State Department of Health (NYSDOH)^51^ categorizes TR as follows, if TR ≤ 10^−6^, then risk is low; if 10^−4^ to 10^−3^ then moderate; if 10^−3^ to 10^−1^ then high; if ≥ 10^−1^ then very high. Likewise, THQ, the TR is also not a specific evaluation of expected cancers. Rather, the upper limit of the likelihood that the individuals may have cancer sometimes in his/her lifetime if the subsequent exposure to that chemical take place^51^.

**Table 4:** Human health risk assessment parameters (EDI, THQ, HI and TR).

Heavy metals	EDI		THQ		HI		TR	
	Adult male	Adult female	Adult male	Adult female	Adult male	Adult female	Adult male	Adult female
Cr	12.5	14.33	4190.78 × 10^–6^	4777.5 × 10^–6^	7443.54 × 10^–6^	8485.68 × 10^–6^	6.28 × 10^–6^	7.16 × 10^–6^
Mn	3.35	3.82	23.94 × 10^–6^	27.3 × 10^–6^			–	–
Fe	18.66	21.27	26.65 × 10^–6^	30.39 × 10^–6^			–	–
Ni	11.63	13.26	581.57 × 10^–6^	663.0 × 10^–6^			1.97 × 10^–7^	2.254 × 10^–7^
Cu	101.28	115.45	2532.0 × 10^–6^	2886.48 × 10^–6^			–	–
Zn	26.58	30.3	88.60 × 10^–6^	101.01 × 10^–6^			–	–

## Conclusion

Present work provides report on the effect of heavy metals overload in *O. niloticus*, especially, in liver and kidney tissues. The results from hematological parameters revealed that exposure of contaminated water may cause respiratory dysfunction as well as compensatory response in fish. Our study strongly supports that contaminated heavy metal caused severe tissue damage, nuclear abnormalities, and DNA damage in liver and kidney in *O. niloticus*. Therefore, nuclear abnormalities and DNA damage could be used as genotoxic biomarkers in response to heavy metals pollution load. Liver seems to be the target organ of these pollutants in *O. niloticus*. In order to have good health of aquatic ecosystems, dwelling fauna and their users, the permissible concentrations of heavy metals in the Yamuna water column and water standards should be regularly monitored.

## Material and methods

### Collection of fish species and water quality analysis

*Oreochromis niloticus* (n = 18) were captured from Yamuna river (Agra, India) [27° 11′2.59″N and 78°1′47.58″E]. Another group of fish (n = 18) were collected from reference water body, Agra, which has no visible point and nonpoint source of pollution. Body length and weight of both groups (polluted and reference) of fish’s were 13.9 ± 1.6 cm, 48 ± 1.4 g and 15.4 ± 2.2 cm and 56 ± 1.5 g respectively. Muscle, liver, and kidney of both groups were acid digested as per the method described by Ref.^3^ for measurement of heavy metals (Fe, Mn, Zn, Cu, Ni, Cr, and Cd). Metal pollution index (MPI) was calculated as below mentioned in the equation^45^:

$${\text{MPI }} = \, \left( {C{\text{m}}_{{1}} \times Cm_{{2}} \times \, \cdots Cm_{n} } \right)^{{{1}/{\text{n}}}}$$

where Cm_1_, Cm_2_ ….. up to Cm_n_ are the concentrations of different metals in the sample. Heavy metals in water were determined by HNO_3_ acid digestion method via Atomic Absorption Spectrometer^55^. Moreover, dissolved oxygen, temperature, conductivity, and pH were measured with the help of digital meters.

### Enzyme assay

Fishes were anesthetized in MS-222 (10%), and blood samples were obtained by cardiac puncture for enzymatic assays such as alkaline phosphatase (ALP), alanine aminotransferase (ALT), aspartate aminotransferase (AST), and creatine kinase (CK). The said enzymatic analysis was performed by ready to use kits (RANDOX Laboratories Ltd., Crumlin United Kingdom) following instructions of the manufacturer.

### Albumin (A) and globulin (G) in serum

Albumin protein was assayed spectrophotometrically with the help of commercial kit (Siemens Ltd., Gujarat, India). Total protein was determined as per the method described by Bradford^56^ with slight modifications and has globulin concentrations after albumin reduction from the total proteins. Albumin to globulin (A:G) ratio was also computed.

### Innate immunity

#### Total leukocyte count (TLC) and differential leukocytes count (DLC)

TLC (10^3^ mm^−3^) was counted with the help of hemocytometer. Blood smear was made and stained with giemsa for DLC counting.

#### Respiratory burst and NOS

Ethylene diamine tetra acetic acid containing blood were assayed for respiratory burst. Nitroblue tetrazolium dye was reduced to formazan during respiratory burst^12,36^. NOS activity was determined as per the method described by Kumar et al.^12^ and Chakrabarti et al.^36^ with some modifications. Tissue homogenate was prepared in phosphate buffer and then centrifuged at 1000×*g* for 10 min. To 100 mL of supernatant, equal volume of Griess reagent was added and incubated at room temperature. The absorbance was recorded at 540 nm.

### Histopathology

Liver and kidney of both groups of fish were removed and after washing in normal saline, fixed in Bouin's fluid as specified by Javed et al.^42^. Ten fish were taken from both exposed and reference group, dissected and slides were prepared in duplicates. After dehydration with different grades of alcohol, double staining were done with hematoxylin and eosin. Prepared slides were then observed under microscope (Leica DM 2500) and were studied for histological changes in exposed tissue as compared to the reference.

### Micronuclei test (MN)

Blood smear was made to study micronuclei (MN) and other nuclear abnormalities in erythrocytes like lobed nuclei, kidney shaped nuclei by the method mentioned in Ahmad and Ahmad^14^. Smeared slides were fixed in 100% methanol, dried, and the stained with giemsa for around 10 min. To calculate the frequencies of different nuclear abnormalities, 1000 erythrocytes were scored from both groups.

### Measurement of DNA damage

DNA damage was measured by single cell gel electrophoresis (SCGE). The SCGE was performed under alkaline condition following the protocol of Singh et al.^57^ with some modification. Liver and kidney slides were scored by using Komet 5.5 imaging system attached with Olympus fluorescent microscope (CX41). DNA damage in terms of migrated tail lengths were measured as the distance between the center of the head and the end of migrated tail in µm.

### Human health risk assessment

#### Estimated daily intake (EDI)

$${\text{EDI}}\,\left({\text{mg}}/{\text{kg body}} - {\text{weight}}/{\text{day}}\right) = {\text{Mc}} \times {\text{IR}}/{\text{Bw}} \times 10^{-3}$$

where Mc is concentration of metal in mg/kg dry weight, IR is ingestion rate (19.5 × 10^−3^ kg/day), Bw is an average body weight for Indian adult male and female are 57 kg and 50 kg respectively^58^.

#### Target hazard quotient (THQ)

It shows non-carcinogenic risk, dimensionless, and was calculated as per USEPA region III risk-based concentration table^59^.

$${\text{THQ }} = \frac{{{\text{Mc}} \times {\text{IR}} \times 10^{ - 3} \times {\text{EF}} \times {\text{ED}}}}{{{\text{RfD}} \times {\text{Bw}} \times {\text{ATn}}}}$$

where EF is exposure frequency (365 days/year), ED is exposure duration (67 years) (in India life expectancy of males = 65 years approx. and for females it is 68 years approx.). Hence, averages of both expectancies have been taken ((https://countryeconomy.com/demography/life-expectancy/india). RfD is reference dose of metals (mg/kg/day) (USEPA^54^) (Table 5). ATn is averaging time for non-carcinogens (365 days/year × ED)^59^.

**Table 5:** Reference dose (RfD) and carcinogenic potency slope oral (CPSo) of heavy metals.

Heavy metals	RfD (mg/kg/day)	CPSo (mg/kg bw-day ^−1^)
Cr	3 × 10^–3^	5 × 10^–1^
Mn	1.4 × 10^–1^	–
Fe	7.0 × 10^–1^	–
Ni	2.0 × 10^–2^	1.7 × 10^–1^
Cu	4.0 × 10^–2^	–
Zn	3.0 × 10^–1^	–

Adapted from USEPA 2011, 2012.

#### Hazard index (HI)

HI was calculated by addition of all THQs (USEPA^59^)

$${\text{HI }} = {\text{ THQCr }} + {\text{THQMn }} + {\text{ THQFe }} + {\text{ THQNi }} + {\text{ THQCu }} + {\text{ THQZn}}$$

#### Target cancer risk (TR)

TR reveals the carcinogenic risk, dimensionless and was determined as per USEPA region III risk-based concentration table^59^.

$${\text{TR }} = \frac{{{\text{Mc}} \times {\text{IR}} \times 10^{ - 3} \times {\text{ CPSo}} \times {\text{EF}} \times {\text{ED}}}}{{{\text{Bw}} \times {\text{ATc}}}}$$

where CPSo is carcinogenic potency slope for oral in mg/kg bw-day^−1^. ATc is averaging time for carcinogens (365 days/year × 67 years), because average life expectancy for Indians (already described earlier).

For heavy metals like Mn, Fe, Cu, and Zn, CPSo have yet not been established^54^ so, TR values for Cr and Ni only was calculated (Table 5).

There are few assumptions one must consider while calculating THQ for human health risk. They are mentioned below:

(a) Ingested dose of pollutant is equal to the absorbed dose^48^.

(b) Cooking has no effect on pollutants^60^.

### Statistical analysis

All the parameters were analyzed in duplicates and values are reported as mean ± standard error mean. Statistical analysis of the data had been performed with Student’s t-test, two-way ANOVA and Duncan’s Multiple Range Test (DMRT) with the help of SPSS software.

### Ethical approval

All applicable international, national, and/or institutional guidelines for the care and use of animals were followed. It was permitted by Ministry of Environment and Forests, Government of India under registration no. 714/02/a/CPCSEA issued and approved by the institutional ethical committee of Department of Biochemistry, Aligarh Muslim University, Aligarh, India.

## Data Availability

Data will be available upon the request to corresponding author.
